# External iliac artery thrombosis after hypogastric artery ligation and pelvic packing for placenta previa percreta

**DOI:** 10.4274/tjod.82642

**Published:** 2018-06-21

**Authors:** Ahmet Rıza Esmer, Reyhan Aslancan, Burak Teymen, Eray Çalışkan

**Affiliations:** 1 Gebze Medical Park Hospital, Clinic of Obstetrics and Gynecology, İzmit, Turkey; 2 Bahçeşehir University Faculty of Medicine, İstanbul, Turkey; 3 Emsey Hospital, Clinic of Cardiology, İstanbul, Turkey; 4 Bahçeşehir University Faculty of Medicine, Department of Obstetrics and Gynecology, İstanbul, Turkey

**Keywords:** Arterial ligation, pelvic packing, thrombosis, placenta previa, placenta percreta

## Abstract

Placenta previa percreta is a serious pregnancy condition that may cause massive bleeding. Life-threatening hemorrhage is commonly managed via cesarean hysterectomy or vascular ligations in order to preserve fertility. We present a case of bilateral external iliac artery thrombosis after pelvic compression and uterine devascularization due to placenta previa percreta. The patient had cesarean section due to ultrasonography and magnetic resonance imaging-diagnosed placenta previa percreta, and stated that she preferred a conservative approach rather than hysterectomy in a case of massive bleeding. Spontaneous hemorrhage was recognized during the operation. Pelvic compression and bilateral uterine and internal iliac artery ligations were performed. The left external iliac artery was accidentally held and bonded as the left internal iliac artery, which was turned loose within a minute after distinguishing the vessels. Emergency angiography that was applied because of patient’s leg pain showed bilateral external iliac artery thrombosis. Angioplasty was performed by a cardiologist for bilateral occlusions. Placenta invasion abnormalities may be managed by pelvic compression or vascular ligations, which have their own serious complications that the surgeon must manage immediately.

## Introduction

Placenta percreta is a serious condition which is managed via pelvic artery ligation, preventive embolization of specific arteries, placental retention, or cesarean hysterectomy^(1)^. Pelvic packing may be considered to support either artery ligation or hysterectomy to reduce an uncontrolled hemorrhage^(2)^. Here we present a case of bilateral external iliac artery thrombosis following ligation of the bilateral hypogastric arteries and a transiently tied left external iliac artery, which underwent immediate angiography and thrombolysis.

## Case Report

**Figure 1:**
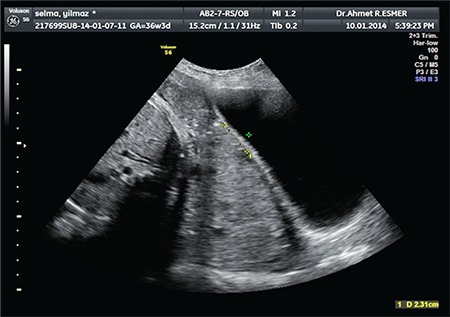
Transabdominal ultrasonographic view of total placenta previa

**Figure 2:**
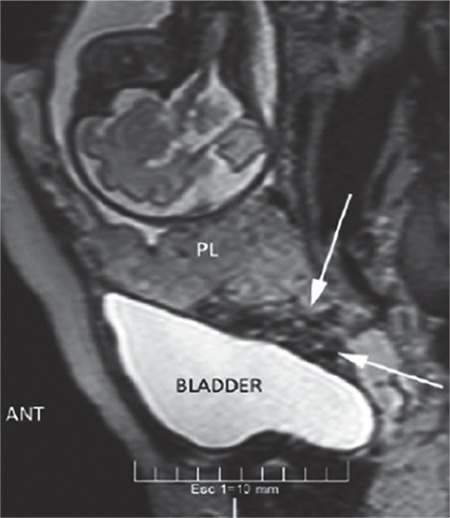
Magnetic resonance imaging of placenta percreta bladder invasion *ANT: Anterior, PL: Placenta*

**Figure 3:**
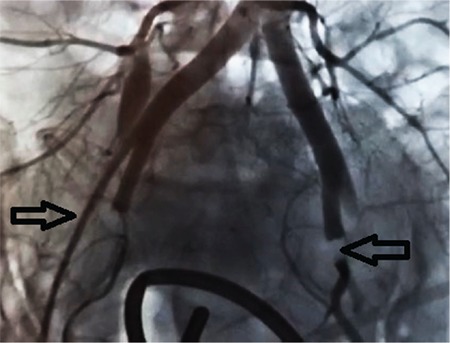
Right and left external iliac arteries. Left external iliac artery failed to show contrast during angiography because of thrombosis, and right external iliac artery was not filled properly with contrast due to thrombosis

A thirty-three-year-old woman was admitted to our clinic at the 37^th^ week of her gestation for delivery with a history of gravidity six, parity two, and abortion three. She had one previous cesarean section delivery. Her preoperative hemoglobin was 10.8 g/dL, prothrombin time (PT) 10.5 seconds, activated partial thromboplastin time (aPTT) 29 seconds, international normalized ratio 2.3, and platelet count 385x10^9^/L. Transabdominal sonography and magnetic resonance imaging displayed total placenta previa, with myometrial invasion to the urinary bladder at the anterior wall of uterus (Figure 1, 2). The patient stated preoperatively that she preferred a conservative approach rather than hysterectomy in case of massive bleeding. After cesarean delivery of a transverse baby through a Pfannenstiel incision and removal of the placenta, a 5-6 cm area of tissue loss was detected at the anterior wall of the uterus and bleeding occurred from the cervix and posterior wall of the bladder. Pelvic packing was applied on the pelvic vessels for 20 minutes, the bleeding sites were sutured with 1.0 polyglactine sutures, and Sengstaken-Blakemore balloon catheter was placed in the uterus before suturing. A stomach balloon was filled with 250 mL saline and an esophageal balloon was filled with 400 mL saline to provide compression on the lower uterine isthmic and cervical bleeds, nevertheless, bleeding continued. Bilateral uterine and hypogastric artery ligations were planned due to hemorrhage. The left external iliac artery was accidentally held and bonded as the left hypogastric artery, which was released within a minute after distinguishing the vessels. Following this mistake, the uterine and hypogastric arteries were ligated on both sides. The patient lost about 2000 cc of blood due to the intraoperative hemorrhage as measured by adding 1650 cc blood in the aspirator and counted gauzes. She received erythrocyte suspension (3 units preoperatively and 4 unites postoperatively) and 3 packs of fresh frozen plasma. The patient had no findings of hypotension or shock at any time. The patient reported severe pain in both legs in the recovery room; it was observed that left dorsalis pedis and femoral artery pulses were absent. Doppler sonography showed a distinct stricture and triphasic flow loss on the left femoral artery. Diagnostic angiography was performed by a cardiologist. After a 6-F introducer sheath was inserted, it was confirmed that both external iliac arteries were occluded (Figure 3). Intravenous heparin (100 IU/kg) was administered afterwards. A 6-F left internal mammary artery catheter was used with 0.035 hydrophilic guide wires to cross the occlusion. Angioplasty was performed first to the right and then contralaterally to the left external iliac artery with a standard balloon (8x80 mm). A completion angiogram concluded the procedure. The femoral access site managed manually with digital pressure. The balloon catheter was removed after 30 hours. The patient and her child were discharged on the 4^th^ postoperative day with no further events. Informed consent was obtained from the patient.

## Discussion

There are mainly two different approaches to placenta implantation abnormalities to prevent excessive blood loss: cesarean hysterectomy or uterus protective techniques. The conservative approach is mainly considered when the patient prefers to spare her uterus for future fertility. Currently, placental invasion abnormalities are managed via radical or staged cesarean hysterectomy, vascular ligations and balloon embolization, placental retention, complex compression hemostasis for which pelvic packing is combined with uterine balloon placement, and partial hysterectomy for focal placental invasions^(3,4)^. The surgeon should be alert to complications of these management techniques. It is reported that common iliac artery thrombosis and acute limb ischemia, unilateral arterial rupture, bilateral pseudoaneurysms, and diminished bilateral leg blood supply due to thrombus and unilateral external iliac artery thrombosis are complications following bilateral hypogastric artery ligation^(5)^. In addition, one case of unilateral external iliac artery thrombosis due to placental retention has been reported^(6)^. Common iliac artery embolization is applied in some cases to prevent excessive hemorrhage due to an abnormally invasive placenta. This method can result in unilateral external iliac artery thromboembolisms and unilateral dorsalis pedis artery thromboembolisms^(7)^. Pelvic packing is an approach to reduce the hemorrhage and helpful in the management of abnormally invasive placenta^(2)^. Although this technique is useful to decrease the bleeding, it may cause significant complications. It is reported that deep vein thrombosis is a possible outcome of pelvic packing due to either pelvic fracture or excessive uterine bleeding^(8)^. There are no reports of arterial thrombosis following pelvic packing. In addition, it is important to note that the swabs used for pelvic packing are generally removed after 36-48 hours via relaparotomy, whereas it was applied for only twenty minutes and removed intraoperatively in our case. According to the patient’s preference, cesarean hysterectomy was avoided in this case. Pelvic packing is selected in conservative techniques and supported by pelvic packing. Since placental retention carries the risk of postoperative hemorrhage and infection, this technique was not preferred for management of the case^(3)^. In our case, it was possible to anticipate left external iliac artery thrombosis because of accidentally holding the external iliac artery during the operation. However, thromboses were observed postoperatively in both the left and right external iliac arteries. This study is the first case showing bilateral external iliac artery thrombosis after the internal iliac artery ligation, and arterial thrombosis after pelvic packing about which the surgeon must suspect and manage immediately via angiography.
